# Modeling the underlying biological processes in Alzheimer's disease using a multivariate competing risk joint model

**DOI:** 10.1002/sim.9425

**Published:** 2022-05-18

**Authors:** Floor M. van Oudenhoven, Sophie H. N. Swinkels, Tobias Hartmann, Dimitris Rizopoulos

**Affiliations:** ^1^ Department of Biostatistics Erasmus MC Rotterdam The Netherlands; ^2^ Department of Epidemiology Erasmus MC Rotterdam The Netherlands; ^3^ Danone Nutricia Research Utrecht The Netherlands; ^4^ German Institute for Dementia Prevention (DIDP) Saarland University Homburg Germany; ^5^ Department of Experimental Neurology Saarland University Homburg Germany

**Keywords:** competing risks, joint model, latent mediation analysis, serial multiple mediator model

## Abstract

Many clinical trials repeatedly measure several longitudinal outcomes on patients. Patient follow‐up can discontinue due to an outcome‐dependent event, such as clinical diagnosis, death, or dropout. Joint modeling is a popular choice for the analysis of this type of data. Using example data from a prodromal Alzheimer's disease trial, we propose a new type of multivariate joint model in which longitudinal brain imaging outcomes and memory impairment ratings are allowed to be associated both with time to open‐label medication and dropout, and where the brain imaging outcomes may also directly affect the memory impairment ratings. Existing joint models for multivariate longitudinal outcomes account for the correlation between the longitudinal outcomes through the random effects, often by assuming a multivariate normal distribution. However, for these models, it is difficult to interpret how the longitudinal outcomes affect each other. We model the dependence between the longitudinal outcomes differently so that a first longitudinal outcome affects a second one. Specifically, for each longitudinal outcome, we use a linear mixed‐effects model to estimate its trajectory, where, for the second longitudinal outcome, we include the linear predictor of the first outcome as a time‐varying covariate. This facilitates an easy and direct interpretation of the association between the longitudinal outcomes and provides a framework for latent mediation analysis to understand the underlying biological processes. For the trial considered here, we found that part of the intervention effect is mediated through hippocampal brain atrophy. The proposed joint models are fitted using a Bayesian framework via MCMC simulation.

## 1 INTRODUCTION

In randomized clinical trials, the focus is often on whether an intervention has a beneficial effect on the outcome of interest rather than the mechanism through which the intervention exerts its effects. However, identifying the mechanism can strengthen the validity of findings and can generate ideas to improve the effectiveness. This is particularly useful for interventions in complex diseases. Alzheimer's disease (AD) is such a complex disease as it is a slowly progressing neurodegenerative disease affecting several domains, including memory, cognition, executive function, and brain atrophy. Consequently, AD trials typically collect several longitudinal outcomes repeatedly measured for months or even years. An example for our work is a long‐term AD trial, namely the LipiDiDiet trial.^1^, ^2^ The LipiDiDiet trial aimed to investigate the effects of medical nutrition (Souvenaid) on cognition and related measures in prodromal AD individuals. Various outcomes, including scores on cognitive performance, memory, and brain atrophy measures, were collected over time. During the trial, subjects that progressed to dementia were allowed to remain in the trial while using open‐label medication, defined as active study product and/or AD medication. However, as the trial was designed to investigate the effects of the intervention on drug‐naïve individuals with prodromal AD, data collected after subjects started open‐label medication were prespecified to be excluded from the efficacy analysis. In addition to the exclusion of data after the start of open‐label medication, some subjects dropped out during the trial.

**FIGURE 1:**
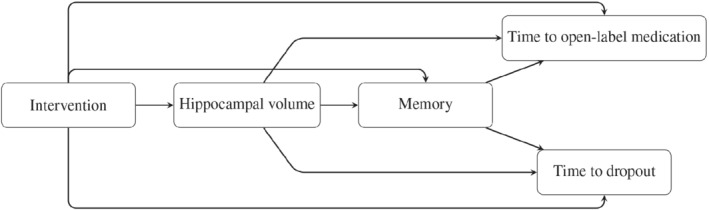
A conceptual diagram of the potential underlying biological processes in the LipiDiDiet data

This article focuses on two longitudinal outcomes: memory domain measured based on a neuropsychological test battery (NTB memory domain) and hippocampal brain atrophy. The hippocampal brain atrophy outcome is expected to affect the memory impairment ratings, as the hippocampus's importance in memory functioning has been demonstrated.^3^ For verbal memory tasks, stronger associations in AD patients have been reported for the volume of the left hippocampus than for the volume of the right hippocampus.^3^, ^4^ Therefore, in this article, we concentrate on the volume of the left hippocampus. The memory impairment ratings, in turn, are expected to affect the time to open‐label medication. Importantly, in this research, we use the time to open‐label medication as a proxy for disease progression. Also, hippocampal brain atrophy might affect the time to open‐label medication, and the same types of relationships might exist for the time to dropout. Furthermore, the intervention is expected to affect both hippocampal brain atrophy and memory. The latter effect might be through hippocampal volume or a different pathway. Figure 1 provides a schematic representation of the potential underlying biological processes in the data. As can be seen, several paths link the intervention to time to open‐label medication and dropout. Additionally, the intervention effect can be partitioned into direct and indirect paths of influence. Our goal is to estimate these different paths to better understand the underlying biological mechanisms, and in particular, to understand how the intervention affects the time to open‐label medication.

Both the use of open‐label medication and dropout resulted in missing data for the longitudinal outcomes. We can expect different (missing) longitudinal trajectories for subjects whose data collected after the start of open‐label medication were censored than for subjects who dropped out of the trial. Both these events, but particularly the use of open‐label medication following progression to dementia, could be related to disease progression. Therefore, the missing data might be nonrandom (i.e., the probability of dropout depends on unobserved longitudinal responses). When analyzing this type of longitudinal data, we rely on methods that jointly estimate the longitudinal outcome and the dropout process, such as the joint model for longitudinal and time‐to‐event data.^5^, ^6^, ^7^ Using the joint modeling framework, it is also possible to deal with the competing events of open‐label medication use and dropout.^8^, ^9^, ^10^ Joint models for two or more longitudinal outcomes have been considered before. For example, Brown et al^11^ proposed a multivariate joint model with a B‐spline formulation for the longitudinal part, and Rizopoulos and Ghosh^12^ proposed a semiparametric multivariate joint model. In these existing joint models for multiple longitudinal outcomes, longitudinal outcomes are assumed to be correlated but not to causally influence one another. Often, in these models, the interdependencies between the longitudinal outcomes are captured by the random effects, typically by assuming a multivariate normal distribution; for other examples, see Chi and Ibrahim^13^ and Andrinopoulou et al.^14^ However, these models are not suitable when in the hypothesized working mechanism one longitudinal outcome is assumed to influence another.

We propose a new type of multivariate competing risk joint model in which, for each of the longitudinal outcomes, we use a linear mixed‐effects model to estimate its evolution over time, where for the second longitudinal outcome, we include the predictor of the first outcome as a time‐varying covariate. The proposed model provides a framework for latent mediation analysis, which we use to estimate the different direct and indirect effects to understand the underlying biological processes. Our approach is different from traditional mediation analysis as we use the predicted values (ie, latent processes) of the longitudinal outcome instead of the observed outcomes.

This article is organized as follows: Section 2 presents the proposed multivariate competing risk joint model. Section 3 describes how the proposed model can be used to investigate mediation. Section 4 shows the results for the proposed model on the LipiDiDiet data. Finally, in Section 5, we evaluate the proposed model's performance.

## 2 MODEL FORMULATION

For subject i (i=1,…,n), let $y_{i1}(t)$ and $y_{i2}(t)$ denote the longitudinal values at time point t for respectively the first and second longitudinal outcome. These values are observed at specific time points that can differ for each subject and each longitudinal outcome. We use mixed‐effects models to describe the trajectories of the longitudinal outcomes over time. In particular, to model the first longitudinal outcome, we postulate

$$\begin{array}{ll} y_{i1}(t) & =m_{i1}(t)+\epsilon_{i1}(t)\;\\ & =x_{i1}^{⊤}(t)\beta_{1}+z_{i1}^{⊤}(t)b_{i1}+\epsilon_{i1}(t),\; \end{array}$$

where $\beta_{1}$ denotes the vector for the fixed regression coefficients, $b_{i1}$ denotes the vector of random effects, $x_{i1}(t)$ and $z_{i1}(t)$ are design vectors for the fixed and random effects, respectively, and $\epsilon_{i1}(t)$ denotes the measurement error term for which we assume $\epsilon_{i1}(t)\;∼𝒩(0,\sigma_{1}^{2})$. Further, the random effects are assumed normally distributed with mean zero and covariance matrix $\sum_{b1}$, being independent of $\epsilon_{i1}(t)$. The linear predictor $m_{i1}(t)$ denotes the true but unobserved trajectory, that is, the latent process, of the first longitudinal outcome over time. We postulate that $m_{i1}(t)$ affects $m_{i2}(t)$, that is, the latent process of the second longitudinal outcome, and we assume that the intervention and other covariates affect both

$$\begin{array}{ll} y_{i2}(t) & =m_{i2}(t)+\epsilon_{i2}(t)\;\\ & =x_{i2}^{⊤}(t)\beta_{2}+z_{i2}^{⊤}(t)b_{i2}+\xi m_{i1}(t)+\epsilon_{i2}(t).\; \end{array}$$

In this way, using the parameter ξ, we can directly interpret the association between the two longitudinal outcomes as it denotes the change in the predicted value of the second longitudinal outcome for a one unit increase in the predicted value of the first longitudinal outcome, holding the other factors constant. Further, $\beta_{2}$, $b_{i2}$, $x_{i2}(t)$, and $z_{i2}(t)$ denote, respectively, the fixed regression coefficients, the random effects, and the design vectors, similar as before but now for the second longitudinal outcome. The random effects $b_{i2}$ are assumed to be normally distributed, with mean zero and covariance matrix $\sum_{b2}$. Note that the random effects $b_{i1}$ and $b_{i2}$ are not assumed to be correlated.

For subject i, let $T_{ik}^{*}$ denote the true event time for event k=1,2, and let $C_{i}$ denote the censoring time. Moreover, let $T_{i}=\min(T_{i1}^{*},T_{i2}^{*},C_{i})$ be the observed survival time and $\delta_{i}$ the event indicator, where $\delta_{i}=k$ in case of the competing events and $\delta_{i}=0$ for censoring. The hazard function for subject i for competing event k is modeled as follows

$$h_{ik}(t,\theta_{sk})=h_{0k}(t)\exp\{\gamma_{k}^{⊤}w_{i}+\alpha_{k1}m_{i1}(t)+\alpha_{k2}m_{i2}(t)\},$$

where $h_{0k}$ denotes the baseline hazard for the kth event, which we approximate in a flexible manner, using B‐splines $\phi_{sk}$. Further, $\theta_{sk}$ is the parameter vector for the kth survival outcome, and $w_{i}$ is a design vector of baseline covariates with a corresponding vector of regression coefficients $\gamma_{k}$. The latent processes of the first and second longitudinal outcome are allowed to be associated with both competing events, quantified by the association parameters $\alpha_{k1}$ and $\alpha_{k2}$.

The parameters are estimated under the Bayesian framework, using Markov chain Monte Carlo (MCMC) methods and using the JAGS software.

## 3 MEDIATION

### 3.1 Different intervention effects

**FIGURE 2:**
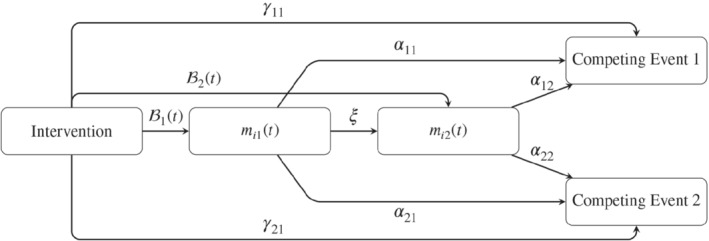
Schematic representation of the proposed joint model. Note that this represents a simplified version of the model, as next to the intervention, also other covariates may affect *m*_*i*1_(*t*), *m*_*i*2_(*t*), and the competing events.

The proposed joint model can be used to understand the process(es) of how the intervention affects the competing risks. A schematic representation of the proposed model is presented in Figure 2, based on which we can distinguish different paths that link the intervention to the competing events, either passing through none, one, or both of the longitudinal outcomes (i.e., their latent processes). Our goal is to estimate each of these paths. For ease of illustration, let ${ℬ}_{1}(t)$ and ${ℬ}_{2}(t)$ denote the regression coefficients involving the intervention effect in, respectively, the first and second longitudinal submodel. These are typically time‐dependent due to the specification of an interaction of intervention by time in the longitudinal models. Based on Figure 2, we can distinguish a direct path, quantified by $\gamma_{11}$, and three different indirect paths linking the intervention to the first competing event, passing through one or both of the longitudinal outcomes. In Supplementary Figures 7a‐c, we highlighted the indirect paths one by one.

The first indirect path we can distinguish (see Supplementary Figure 7a) is the path that links the intervention to the first event, passing through the first longitudinal outcome. The parameter ${ℬ}_{1}(t)$ describes the intervention effect on the first longitudinal outcome over time, and the parameter $\alpha_{11}$ quantifies the effect of this longitudinal outcome on the first event. Therefore, the indirect effect of the intervention on the first event mediated through the first longitudinal outcome is quantified as the product $\alpha_{11}{ℬ}_{1}(t)$.

Second, we observe an indirect path (see Supplementary Figure 7b) quantifying the intervention effect on the first event mediated through the second longitudinal outcome. This path involves the parameters ${ℬ}_{2}(t)$ and $\alpha_{12}$.

The third indirect path (see Supplementary Figure 7c) quantifies the mediation through the first and second longitudinal outcome on the first competing event. That is, the intervention may affect the first longitudinal outcome, quantified by the parameter ${ℬ}_{1}(t)$, which in turn is expected to affect the second longitudinal outcome. The second longitudinal outcome, in turn, may affect the risk of the first event. This path is quantified as $\xi \;\alpha_{12}\;{ℬ}_{1}(t)$. As is the case for the Cox model, these quantities' exponent expresses the effects as multiplicative changes on the hazard. For example, $\exp\{\xi \;\alpha_{12}\;{ℬ}_{1}(t)\}$ denotes the increase in the risk of the first event due to the indirect effect of the intervention through both longitudinal outcomes.

Lastly, the total intervention effect on the first event in the joint model is the hazard ratio of the active vs the control group, with all other covariates held constant. For the proposed joint model, this hazard ratio is a combination of the direct path, which is time‐independent, and the three, typically time‐dependent, indirect paths. Therefore for the proposed joint model, the time‐varying hazard ratio for the overall intervention effect can be expressed as:

$$𝒯(t)=\exp\{\gamma_{11}+\alpha_{11}{ℬ}_{1}(t)+\alpha_{12}{ℬ}_{2}(t)+\xi \alpha_{12}{ℬ}_{1}(t)\}.$$

Note that the hazard ratios for the direct, indirect, and total effect are conditional on the random effects and therefore have subject‐specific interpretations. For more information and a method to obtain the marginal overall treatment effect, we refer to Reference 15.

### 3.2 Traditional mediation analysis and our approach

There are two major general approaches for calculating the point estimate of the mediated effect in single‐mediator models: the difference in coefficients and the product of coefficients methods. Consider the following three regression equations:

$$\begin{array}{ll} Y & =i_{1}+cX+\epsilon_{1},\;\\ Y & =i_{2}+c^{\prime }X+bM+\epsilon_{2},\;\\ M & =i_{3}+aX+\epsilon_{3},\; \end{array}$$

where X is the independent variable, M is the mediator, Y is the dependent variable, c represents the relation between the independent and dependent variable, $c^{\prime }$ represents the relation between the independent and dependent variables adjusted for the effect of the mediator, b represents the relation between the mediator and the dependent variable adjusted for the effect of the independent variable, and a represents the relation between the independent variable and the mediator. With the difference in coefficients methods, the difference between the regression coefficients before and after adjustment for the mediator is calculated: $c-c^{\prime }$. The product of coefficients method computes the product ab and is based on the rationale that the mediated effect is equal to the effect of the independent variable on the mediator times the effect of the mediator on the dependent variable. MacKinnon et al^16^ have shown that the two estimators, $c-c^{\prime }$ and ab, are mathematically equivalent when the dependent variable is continuous and ordinary regression is used. However, for multilevel models, logistic or probit regression, and survival analysis, the estimators are not always equivalent.^16^, ^17^, ^18^, ^19^

**FIGURE 3:**
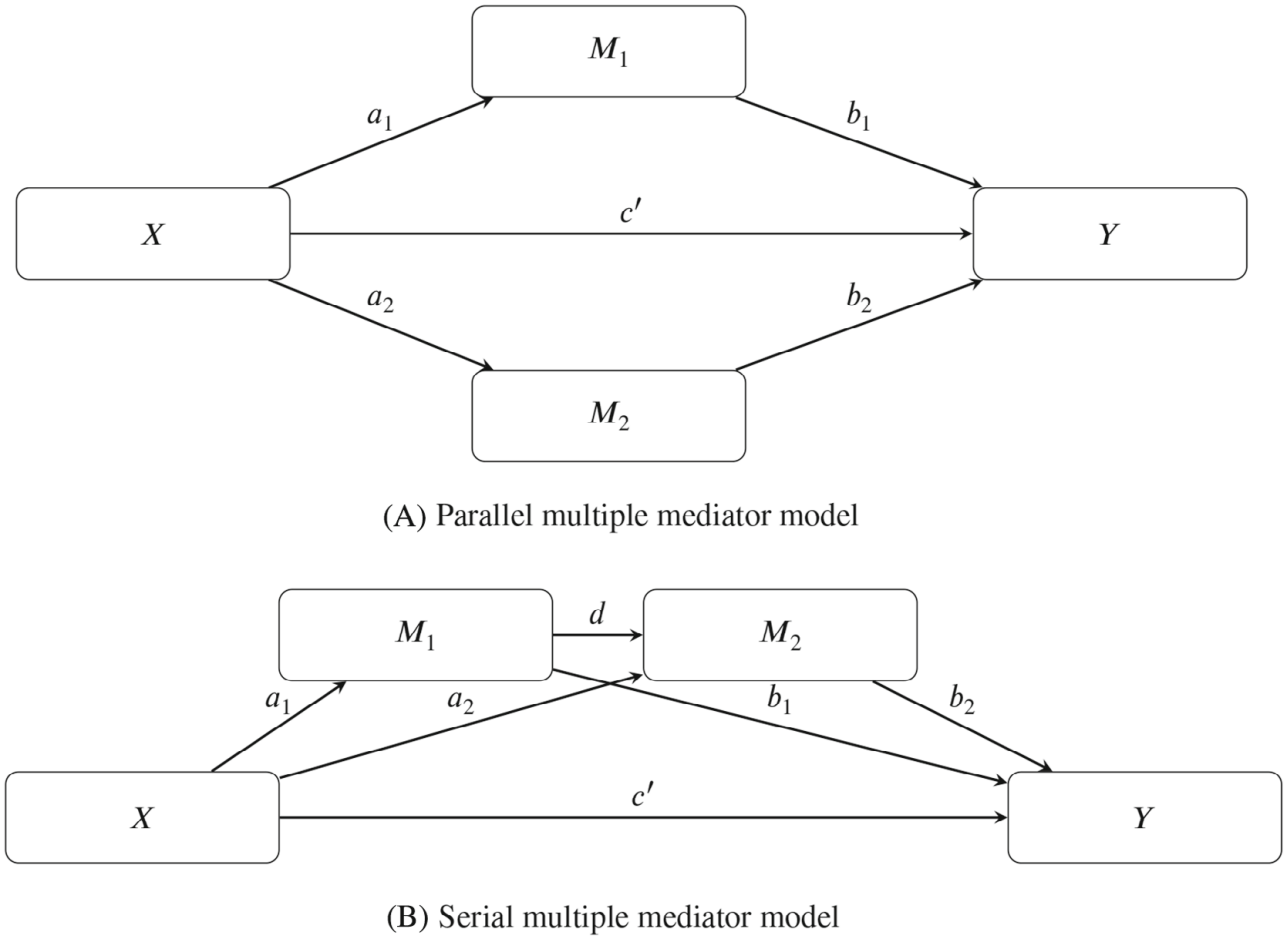
Two multiple mediator models with two mediators

The traditional single‐mediator model can be extended to models with more than one mediator. Two forms of multiple mediator models are serial multiple mediator models, in which the mediators are linked together in a causal chain, and parallel multiple mediator models, in which the mediators are merely allowed to correlate but not to causally influence another mediator in the model.^20^ The parallel and serial mediator model are shown in Figure 3. The existing multivariate joint models belong to the class of parallel multiple mediator models. In these models, there is no direct link between the mediators; the mediators are allowed to correlate but not to causally influence one another.

This article's proposed model has the form of the serial multiple mediator model with two mediators. As can be seen in the lower panel of Figure 3, X is modeled as affecting Y through one direct and three indirect paths. The four regression equations that represent the two‐mediator serial mediator model are:

$$\begin{array}{ll} Y & =i_{1}+cX+\epsilon_{1},\;\\ Y & =i_{2}+c^{\prime }X+b_{1}M_{1}+b_{2}M_{2}+\epsilon_{2},\;\\ M_{1} & =i_{3}+a_{1}X+\epsilon_{3},\;\\ M_{2} & =i_{4}+a_{2}X+dM_{2}+\epsilon_{4},\; \end{array}$$

where the total indirect effect can either be calculated by the difference in coefficients method, quantified as $c-c^{\prime }$, or by the product of coefficients methods, quantified as the sum of the three indirect effects $a_{1}b_{1}$, $a_{2}b_{2}$, and $a_{1}db_{2}$.

Although the proposed model has the form of the serial multiple mediator model, our approach to assessing latent mediation analysis differs from traditional mediation analysis for a few reasons. First, we assume that the observed longitudinal outcomes are realizations with error from latent processes, and we postulate that the latent process of the first longitudinal may affect the latent process of the second longitudinal outcome. Using the latent processes is advantageous as it allows to distinguish the true process of interest from its noisy observations. For the estimation of the mediated effect on the risk of the first competing event through the longitudinal outcomes, based on the product of coefficients method, we need the following equation:

$$h_{i1}(t)=h_{01}(t)\exp\{\gamma_{11}^{\prime }w_{i}+\alpha_{11}m_{i1}(t)+\alpha_{12}m_{i2}(t)\}.$$

The total indirect effect based on the product of coefficients methods can be calculated by taking the sum of the three indirect effects $\alpha_{11}{ℬ}_{1}(t)$, $\alpha_{12}{ℬ}_{2}(t)$, and $\xi \alpha_{12}{ℬ}_{1}(t)$. As such, another advantage of using a joint model compared to traditional mediation analysis is that the path coefficients for the product of coefficients method can be estimated based on a single equation. In other words, they can be estimated simultaneously based on the same model while controlling for one another.

To estimate the mediated effect based on the difference in coefficients method, we would need a second equation to obtain the unadjusted effect of the intervention on the risk of the first event ($\gamma_{11}$). For this reason, we do not recommend using the difference in coefficients method for our approach, as it is unclear whether we can combine estimates based on different models in this setting. Furthermore, modeling an interaction of the intervention by time in the longitudinal submodel(s) results in a time‐dependent intervention effect on the survival outcome. Using the difference in coefficients method it is not possible to capture such a time‐dependent mediated effect, as the estimator ($\gamma_{11}-\gamma_{11}^{\prime }$) is time‐independent by definition. Using the product of coefficients methods, it is straightforward to estimate a time‐dependent mediated effect because the estimator $\alpha_{11}{ℬ}_{1}(t)+\alpha_{12}{ℬ}_{2}(t)+\xi \alpha_{12}{ℬ}_{1}(t)$ includes the time‐dependent intervention effects on the longitudinal outcomes ${ℬ}_{1}(t)$ and ${ℬ}_{2}(t)$.

## 4 ANALYSIS OF THE LIPIDIDIET DATA

This section presents the analysis of the LipiDiDiet trial, briefly introduced in Section 1, using the proposed multivariate competing risk joint model. The LipiDiDiet trial is a randomized, controlled, multicenter trial performed at study sites in Finland, Germany, the Netherlands, and Sweden. The trial had an initial 24‐month intervention period and up to 72‐month double‐blind extension periods and showed positive effects on longitudinal measures of cognition, functioning, and brain atrophy.^1^, ^2^ Here, we focus on the first 36 months of intervention, which currently is the maximum reported intervention period. Our primary interest is in estimating the different effects of how the intervention affects the risk of starting open‐label medication use, both directly and indirectly, through one or both of the longitudinal outcomes. In the model, we assume that there is a true underlying profile for each subject, that is, the latent process $m_{i1}(t)$ that dictates how the observed values of hippocampal volume change over time. Likewise, we assume that the NTB memory domain instrument measures the true underlying memory profile $m_{i2}(t)$. We model that the latent process of hippocampal volume $m_{i1}(t)$ has a direct effect on the latent process of NTB memory domain $m_{i2}(t)$. We assume that the intervention and other covariates affect both latent processes, and we allow both latent processes to be associated with the risks of open‐label medication and dropout. To model hippocampal volume, we use a mixed‐effects submodel with random intercepts and slopes. We include both a main effect of the intervention and an interaction of intervention by time to allow the trajectory of the intervention groups to be different over time. Further, following References 1 and 2, we correct for the effect of baseline MMSE and site. The mixed‐effects submodel for hippocampal volume is

$$\begin{array}{llll} y_{i1}(t) & =m_{i1}(t)+\epsilon_{i1}(t)\; & & \\ & =\beta_{10}+\beta_{11}t+\beta_{12}{\text{intervention}}_{i}+\beta_{13}{\text{intervention}}_{i}\times t+\beta_{14}{\text{bmmse}}_{i}+\beta_{15}{\text{site}}_{i}+b_{i10}+b_{i11}t+\epsilon_{i1}(t).\; & & \end{array}$$

The model for NTB memory domain takes the same form, except we include the latent process of hippocampal volume as a time‐varying covariate.

$$\begin{array}{llll} y_{i2}(t) & =m_{i2}(t)+\epsilon_{i2}(t)\; & & \\ & =\beta_{20}+\beta_{21}t+\beta_{22}{\text{intervention}}_{i}+\beta_{23}{\text{intervention}}_{i}\times t+\beta_{24}{\text{bmmse}}_{i}+\beta_{25}{\text{site}}_{i}\; & & \\ & \;+\xi m_{i1}(t)+b_{i20}+b_{i21}t+\epsilon_{i2}(t).\; & & \end{array}$$

For these models, ${ℬ}_{1}(t)$ and ${ℬ}_{2}(t)$, defined as the regression coefficients involving the intervention effects denote $\beta_{12}$ and $\beta_{13}$ for the first longitudinal submodel and $\beta_{22}$ and $\beta_{23}$ for the second longitudinal submodel. To facilitate the comparison among the longitudinal outcomes, we scaled both longitudinal outcomes to zero‐mean and unit variances using the mean and standard deviation (SD) among all subjects and time points.

To model the two competing events, we use cause‐specific hazard regression with separate Cox proportional hazards models for each of the competing events. We include the intervention, baseline MMSE, and site as baseline covariates (with k=1 for open‐label medication and k=2 for dropout)

$$h_{ik}(t,\theta_{sk})=h_{0k}(t)\exp\{\gamma_{k1}{\text{intervention}}_{i}+\gamma_{k2}{\text{bmmse}}_{i}+\gamma_{k3}{\text{site}}_{i}+\alpha_{k1}m_{i1}(t)+\alpha_{k2}m_{i2}(t)\}.$$

The association parameters $\alpha_{k1}$ and $\alpha_{k2}$ measure the strength of the association between the longitudinal outcomes and the risk of the corresponding events. For example, the quantity $\exp(\alpha_{11})$ denotes the hazard ratio for open‐label medication use at time point t for one SD increase in the longitudinal trajectory of hippocampal volume at the same time point. We ran the MCMC for 100 000 iterations and discarded the first 50 000 iterations as burn‐in. Detailed information on the estimation procedure, such as the prior distributions, can be found in the supplementary material. Convergence was assessed based on trace plots, based on which we concluded that the fit is satisfactory.

Next to the different mediated effects, we are also interested in the overall intervention effect on the risk of open‐label medication use. Here, the overall intervention effect 𝒯(t) can be expressed as:

$$𝒯(t)=\exp\{\gamma_{11}+\alpha_{11}\;(\beta_{12}+\beta_{13}t)+\alpha_{12}\;(\beta_{22}+\beta_{23}t)+\xi \;\alpha_{12}\;(\beta_{12}+\beta_{13}t)\}.$$

Table 1 shows the results using the proposed multivariate joint model with hippocampal volume and NTB memory domain as longitudinal outcomes. Only the results for the most relevant parameters are shown here (all results are shown in the supplementary material).

**TABLE 1:** Posterior means, standard deviation, and the 95% credibility intervals for the multivariate joint model using left hippocampal volume and NTB memory domain as longitudinal outcomes

	Mean	SD	95% credibility interval
*Longitudinal process (hippocampal volume)*			
$\beta_{11}$	−0.183	0.021	−0.224 to −0.143
$\beta_{12}({ℬ}_{1}(t))$	−0.064	0.118	−0.296 to 0.166
$\beta_{13}({ℬ}_{1}(t))$	0.071	0.030	0.011 to 0.129
*Longitudinal process (NTB memory domain)*			
$\beta_{21}$	−0.088	0.034	−0.155 to −0.022
$\beta_{22}({ℬ}_{2}(t))$	0.060	0.091	−0.119 to 0.239
$\beta_{23}({ℬ}_{2}(t))$	0.051	0.046	−0.038 to 0.140
ξ	0.354	0.057	0.244 to 0.465
*Survival process (open‐label medication)*			
$\gamma_{11}$	0.153	0.210	−0.259 to 0.563
$\alpha_{11}$	−0.416	0.151	−0.710 to −0.118
$\alpha_{12}$	−1.073	0.166	−1.409 to −0.758
*Survival process (dropout)*			
$\gamma_{21}$	−0.241	0.179	−0.597 to 0.114
$\alpha_{21}$	0.047	0.142	−0.230 to 0.327
$\alpha_{22}$	−0.242	0.136	−0.509 to 0.025

The parameter $\gamma_{11}$ quantifies the direct effect on the risk of open‐label medication use. However, the 95% credible interval for $\gamma_{11}$ does include 0, indicating there is much uncertainty about this effect.

**FIGURE 4:**
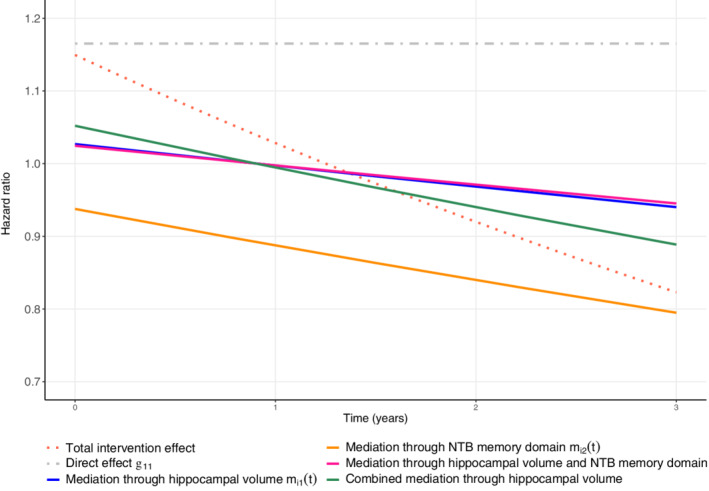
The total intervention effect, the direct effect, and the different mediated effects for the LipiDiDiet data. The colors correspond with the mediation paths in Figure 7a‐c. The combined mediation through hippocampal volume denotes the total mediation through hippocampal volume. It is the sum of the mediation through only hippocampal volume and through both hippocampal volume and NTB memory domain. Further, the total intervention effect is the sum of the direct effect and the different mediated effects

For the first indirect path (see Supplementary Figure 7a), that is, the path that quantifies the intervention effect on open‐label medication mediated through hippocampal volume, we observe a strong intervention effect on hippocampal volume ($\beta_{13}$). In particular, we observe 0.071 SD (95% CI: 0.011 to 0.129) less reduction in hippocampal volume per year in the active group than in the control group. Furthermore, we observe a strong association ($\alpha_{11}$) between hippocampal volume and the risk of open‐label medication. The sign of the α‐coefficient indicates the direction of the association. For hippocampal volume, the sign of the coefficient is negative, indicating that a higher value is found to be associated with a lower risk of open‐label medication use. Specifically an increase of one SD (0.62 units) in the trajectory of hippocampal volume is estimated with a risk reduction of 34% for open‐label medication use ($\left(1-\exp(\alpha_{11}\right)$ = 0.34). The effect of the intervention on open‐label medication mediated through this path expressed as the time‐varying hazard ratio is shown in Figure 4. This figure also shows the results of the other mediated effects and the total intervention effect.

The second indirect path (see Supplementary Figure 7b) quantifies the intervention effect on open‐label medication through NTB memory domain. In this path, the parameter $\beta_{23}$ describes the intervention effect on NTB memory domain over time. The average decrease is estimated to be 0.051 SD (95% CI: −0.038 to 0.140) per year less in the active group than in the control group. Importantly, this effect does not quantify the full treatment effect on NTB memory as part of the effect is mediated through hippocampal volume. In its turn, the predicted value of NTB memory domain is strongly associated with the risk of open‐label medication, quantified by the parameter $\alpha_{12}$. Also for NTB memory domain, a higher value is associated with a lower risk of open‐label medication use. Specifically, an increase of one SD (0.90 units) in the trajectory of NTB memory is estimated with a risk reduction of 66.0% ($\left(1-\exp(\alpha_{12}\right)$ = 0.66).

The parameter ξ reflects the association between the two longitudinal outcomes. We observe a strong association; an increase of one SD (0.62 units) in the trajectory of hippocampal volume is estimated to increase the trajectory of NTB memory domain with 0.354 SD (95% CI: 0.244 to 0.465).

## 5 SIMULATION STUDY

We performed four simulation studies. The first simulation study aimed to verify that the proposed method results in unbiased estimates. In the second simulation study, we investigated the bias when the actual underlying data mechanism was based on the proposed model, with a first longitudinal outcome affecting a second one, but when only one of them was taken into account. Specifically, we used a univariate competing risk joint model for the first longitudinal outcome in the analysis step. In the third and fourth simulation study, we respectively compared the proposed model with the existing multivariate joint model and investigated the robustness of the direct and indirect effects when the model is slightly misspecified. For information on these simulation studies, we refer to the supplementary material.

### 5.1 Design

Data for the first three simulation studies were simulated under the same model structure, which we will now describe. For each simulation study and each scenario, we simulated 200 datasets based on the proposed multivariate competing risk joint model. The longitudinal submodels included an intercept, a linear time effect, an interaction of treatment by time, and random intercepts and slopes. In the second longitudinal submodel, we included the linear predictor of the first outcome as a time‐varying covariate, where the parameter ξ quantified the association between the longitudinal outcomes. For the first and third simulation study we used ξ=−0.5. For the second simulation study, we considered four simulation scenarios, for which we used parameter values for ξ with an increasing magnitude: (I) ξ=−0.01, (II) ξ=−1, (III) ξ=−2, and (IV) ξ=−3. For the survival submodels, we used cause‐specific hazard regression with separate Cox proportional hazards models with the intervention as the only covariate. In the first three simulation studies, the remaining parameters were $\beta_{10}=0.07$, $\beta_{11}=-0.25$, $\beta_{12}=0.12$, $\beta_{20}=0.15$, $\beta_{21}=0.45$, $\beta_{22}=-0.10$, $\gamma_{11}=-0.2$, $\gamma_{21}=0.1$, $\alpha_{11}=-0.6$, $\alpha_{12}=0.4$, $\alpha_{21}=-0.1$, $\alpha_{22}=0.1$. The baseline hazards were simulated using B‐splines, and we used the same knots and spline coefficients for both survival submodels. For each dataset, we simulated 500 subjects and 10 equally spaced measurements per subject. The maximum follow‐up was 3.2, and the mean of the exponential distribution for the censoring mechanism was 5.

### 5.2 Analysis and results

#### 5.2.1 Simulation study 1

In the first simulation study, we analyzed each simulated dataset with the same model structure as in the simulation step. The results are shown in Table 2, where the mean, bias, and coverage rate are given for each parameter. The bias is the mean difference between the estimate of the simulation and the true value. The coverage rate is the percentage of times the true parameter value falls in each simulation's credible interval. The proposed model seems to be performing well as means are close to the true values, the bias is small, and coverage rates are high. Supplementary Figure 9 also compares the average overall intervention effect with the true intervention effect. As can be seen, they are nearly identical.

**TABLE 2:** Results of simulation study 1 including the true parameter values, the mean of the MCMC sample means, the bias, and coverage for each parameter

	True values	Mean	Bias	Coverage
$\beta_{10}$	0.070	0.066	−0.004	94%
$\beta_{11}$	−0.250	−0.251	−0.001	97%
$\beta_{12}$	0.120	0.120	0.000	98%
$\beta_{20}$	0.150	0.149	−0.001	94%
$\beta_{21}$	0.450	0.452	0.002	96%
$\beta_{22}$	−0.100	−0.100	0.000	96%
$\gamma_{11}$	−0.200	−0.231	−0.031	95%
$\gamma_{21}$	0.100	0.085	−0.015	97%
$\alpha_{11}$	−0.600	−0.599	0.001	96%
$\alpha_{21}$	−0.100	−0.096	0.004	95%
$\alpha_{12}$	0.400	0.390	−0.010	95%
$\alpha_{22}$	0.100	0.093	−0.007	94%
ξ	−0.500	−0.497	0.003	95%

*Note*: Results are based on 200 simulated datasets.

#### 5.2.2 Simulation study 2

**FIGURE 5:**
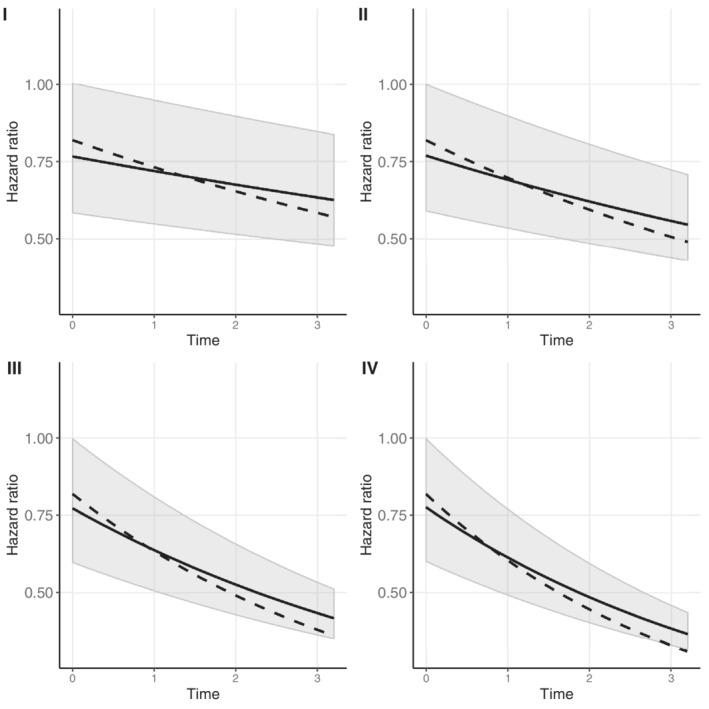
Average time‐varying overall intervention effect 𝒯(t) (solid line) based on 200 simulated datasets with corresponding 95% percentile confidence interval for simulation scenarios I) *ξ* = −0.01, II) *ξ* = −1, III) *ξ* = −2, and IV) *ξ* = −3. The dashed line denotes the true overall intervention effect

In the second simulation study, we analyzed each simulated dataset with a model in which the second longitudinal outcome was not included. We simulated the data under the proposed multivariate competing risk model but analyzed the data with a univariate competing risk joint model for the first longitudinal outcome. Supplementary Figure 8 presents a visualization of this simulation study. The gray parts denote the parameters that are omitted from the model. The results for each simulation scenario are presented in Figure 5. As can be seen, the overall intervention effect as estimated by the univariate joint model is biased in this situation, and the bias increases for larger values of ξ.

## 6 DISCUSSION

Motivated by data from a prodromal AD trial, we proposed a multivariate competing risk joint model in which a first longitudinal outcome is allowed to influence a second longitudinal outcome. Existing multivariate joint models have the form of so‐called parallel mediator models, in which the longitudinal outcomes are merely allowed to be correlated but not to causally influence one another. Using the proposed model, we could model a direct effect of hippocampal brain atrophy on the memory impairment ratings. We allowed both hippocampal brain atrophy and memory to be associated with the risk of open‐label medication use and dropout. The intervention was also allowed to affect both hippocampal brain atrophy and memory, with the latter effect being direct or indirect through hippocampal volume. The proposed method provided a framework for latent mediation analysis, which helped understand the intervention's underlying biological processes. For the LipiDiDiet trial, we found that part of the intervention effect is mediated through hippocampal brain atrophy. However, there could be other effects of the underlying biological processes that we were not aware of, and therefore did not consider in our model.

For the data we considered here, the choice on the directionality was based on biological grounds. As the intervention (Souvenaid) was designed to provide neuroprotection (by supplying rate‐limiting compounds for brain phospholipid synthesis and addressing multiple AD‐related pathological processes^1^), our hypothesized working mechanism was that the intervention affects the brain, which is noticeable with brain imaging outcomes of, for example, hippocampal volume. In turn, changes in the brain may affect how well patients perform on cognitive tests, such as on NTB memory domain. For other datasets where the predominant order between the longitudinal outcomes might be less clear, a decision on the directionality should be made with care and together with a clinical expert. However, we also argue that the proposed model is developed explicitly for situations in which the longitudinal outcomes are hypothesized to be linked together in a causal chain, where a first longitudinal outcome is expected to affect a second one. If no direct link or clear order between the outcomes is assumed, the existing multivariate joint models are perhaps more suitable as the outcomes are only allowed to be correlated in these models. Also, the estimation process here cannot be entirely separated for the two longitudinal outcomes due to their simultaneous estimation (for more information, we refer to the supplementary material).

A first simulation study showed good performance of the proposed method. A second simulation study showed the bias in the overall intervention effect when the data was analyzed with a standard univariate competing risk joint model, while the actual underlying data mechanism was based on the proposed model.

We argue that the joint model, and particularly the proposed type of joint model, is a very suitable and intuitive method for latent mediation analysis with a survival outcome and has certain advantages compared to traditional mediation analysis. First, the different effects that form the total indirect effect, that is, the total mediated effect, can be estimated based on the same joint model while controlling for one another. Second, it is straightforward to model a time‐varying effect on the survival outcome using a joint model. Third, in the proposed method, we assume that the latent process rather than the observed values of the first longitudinal outcome affects the latent process of the second longitudinal outcome, which allows us to focus on the true process of interest instead of its noisy observations. This is different from traditional mediation analysis, where the observed outcomes are used. Another difference is that the hazard ratios for the direct, indirect, and total effect presented here are conditional hazard ratios, whereas often marginal hazard ratios are considered.

In this article, we have assumed that the first longitudinal outcome's underlying value at time t affects the second longitudinal outcome's underlying value at the same time point. However, similar to the association structure between the longitudinal outcome and the survival outcome,^21^, ^22^ we could use a more complex association structure for the association between the longitudinal outcomes. For example, the second longitudinal outcome's value at time point t could be related to the slope at or the cumulative value up to t of the first longitudinal outcome. A simulation study (not shown here) confirmed that the proposed method also works for these types of dependencies between the longitudinal outcomes.

Checking for model misspecification is always important, but it is even more important for our approach as the latent process of the second longitudinal outcome (i.e., NTB memory) depends on the model for the first longitudinal outcome and whether this model is correctly specified. It is recommended to investigate the model's fit using figures, such as comparing the fitted vs predicted trajectories for different subjects and examining the models' assumptions using QQ and residuals vs fitted values plots. These figures can often show if a model adequately fits the data and guide analysts to adapt it if it does not fit the data. Here, we have used linear models for both longitudinal outcomes, but it is also possible to use more complex functions of time such as quadratic or higher‐order polynomials, such as splines. We suggest fitting several plausible models as a sensitivity analysis. In the supplementary material, we included such a sensitivity analysis for the data considered in this article.

We modeled a constant intervention effect across subjects and across time within subjects. One should be aware that this is a simplification that might be more or less permissible depending on the nature of the intervention, disease, and time frame under investigation. However, when this assumption is not plausible, the model could easily be extended with a random treatment effect and a random treatment by time interaction (and potentially more complex forms of this interaction), allowing each subject to respond differently to the treatment, that is, having its own time‐varying treatment effect.

Finally, it is important to emphasize that although we have been able to perform latent mediation analysis, we did not prove the pattern of causation, as shown in Figure 1. One of the main assumptions in causal mediation analysis is the assumption of sequential ignorability, which, for a single mediator, assumes that (i) given the observed covariates, the intervention assignment is ignorable of the mediator process and the outcome of interest, and (ii) given the observed covariates and the intervention, the mediator is ignorable of the outcome. Usually, the randomization can only guarantee the ignorability of the intervention assignment, whereas the second part of this assumption also requires that, conditional on the intervention and the observed covariates, the mediator can be treated as if it were randomized. In other words, the ignorability of the mediator implies that there is no unmeasured confounding between the mediator and the outcome, which excludes the possibility of both unobserved pretreatment confounding or any post‐treatment confounding. In our case, with multiple (causally) dependent mediators, this ignorability assumption is violated as one mediator acts as a post‐treatment confounder for the other mediator on the outcome of interest.^23^, ^24^ Moreover, because the mediators are repeatedly measured, the association between a mediator at a given time point and the outcome may be confounded by other previously measured assessments of the mediator.^25^ It is of our future interest to investigate how we can extend the proposed model to allow for a causal interpretation. However, even for the joint model with one longitudinal outcome, research on its causal (latent) mediation mechanism is very limited^26^, ^27^ and has yet to be investigated.

The code to fit the proposed multivariate competing risk joint model is available in the supplementary material.

## CONFLICT OF INTEREST

The authors Floor M. van Oudenhoven and Sophie H. N. Swinkels are employees of Danone Nutricia Research.

## Supporting information


**Data S1** Supplementary material

## Data Availability

The data are proprietary information of Danone Nutricia Research.
