# Supplementary material for: Modeling the underlying biological processes in Alzheimer's disease using a multivariate competing risk joint model
Source: Stat Med. 2022 May 18;41(17):3421–33. doi: 10.1002/sim.9425 (PMC9545329; doi:10.1002/sim.9425)
Supplement: Supplementary file 1 — Data S1 Supplementary material [file SIM-41-3421-s001.pdf]

Supporting information for Modeling the underlying biological processes in Alzheimer's disease using a multivariate competing risk joint model

Floor M. van Oudenhoven[1,2,3], Sophie H. N. Swinkels[3], Tobias Hartmann[4,5] and Dimitris Rizopoulos[1,2]

1. Department of Biostatistics, Erasmus MC, Rotterdam, The Netherlands
2. Department of Epidemiology, Erasmus MC, Rotterdam, The Netherlands
3. Danone Nutricia Research, Utrecht, The Netherlands
4. German Institute for Dementia Prevention (DIDP), Saarland University, Germany
5. Department of Experimental Neurology, Saarland University, Germany

1 Estimation

We use the Bayesian framework to estimate the proposed joint model and derive the parameter's posterior distributions using a Markov chain Monte Carlo (MCMC) algorithm. The contribution for the ith subject to the posterior distribution of the joint model can be formulated as follows

$$p(\boldsymbol{\theta}, \boldsymbol{b}_i \mid T_i, \delta_i, \boldsymbol{y}_{i1}, \boldsymbol{y}_{i2}) \propto p(T_i, \delta_i \mid \boldsymbol{b}_{i1}, \boldsymbol{b}_{i2}, \boldsymbol{\beta}_1, \boldsymbol{\beta}_2, \boldsymbol{\theta}_s) p(\boldsymbol{y}_{i1} \mid \boldsymbol{b}_{i1}, \boldsymbol{\theta}_{y1}) p(\boldsymbol{y}_{i2} \mid \boldsymbol{b}_{i1}, \boldsymbol{b}_{i2}, \boldsymbol{\theta}_{y1}, \boldsymbol{\theta}_{y2}) \times \\ p(\boldsymbol{b}_{i1} \mid \boldsymbol{\theta}_{y_1}) p(\boldsymbol{b}_{i2} \mid \boldsymbol{\theta}_{y_1}, \boldsymbol{\theta}_{y_2}) p(\boldsymbol{\theta}_{y1}) p(\boldsymbol{\theta}_{y2}) p(\boldsymbol{\theta}_s),$$

where $\boldsymbol{\theta} = (\boldsymbol{\theta}_{y1}^\top, \boldsymbol{\theta}_{y2}^\top, \boldsymbol{\theta}_{s1}^\top, \boldsymbol{\theta}_{s2}^\top)^\top$ denotes the parameter vector for the longitudinal and the survival outcomes. The parameter vector for the kth survival outcome (for k = 1,2) denotes $\boldsymbol{\theta}_{sk} = (\boldsymbol{\phi}_{sk}^\top, \boldsymbol{\gamma}_k^\top, \alpha_{k1}, \alpha_{k2})^\top$. The parameter vector $\boldsymbol{\theta}_{y2}$ also contains ξ.

The likelihood contribution of subject i to the survival part is written as

$$p(T_i, \delta_i \mid \boldsymbol{b}_i, \boldsymbol{\beta}_1, \boldsymbol{\beta}_2, \boldsymbol{\theta}_s) = \prod_{k=1}^{K} [h_0(T_i \mid \boldsymbol{\phi}_{sk}) \exp\{\boldsymbol{\gamma}_k^\top \boldsymbol{w}_i + \alpha_{k1} m_{i1}(T_i) + \alpha_{k2} m_{i2}(T_i)\}]^{I(\delta_i = k)} \times \\ \exp\Big\{ -\sum_{k=1}^{K} \int_0^{T_i} h_0(s \mid \boldsymbol{\phi}_{sk}) \exp\{\boldsymbol{\gamma}_k^\top \boldsymbol{w}_i + \alpha_{k1} m_{i1}(s) + \alpha_{k2} m_{i2}(s)\} ds \Big\},$$

and the likelihood contribution to the longitudinal part is given by

$$
\begin{aligned}
p(\boldsymbol{y}_{i1}, \boldsymbol{y}_{i2} \mid \boldsymbol{b}_i, \boldsymbol{\theta}_{y1}, \boldsymbol{\theta}_{y2}) = & \frac{1}{\sqrt{2\pi\sigma_2}} \exp\Big\{ -\frac{1}{2\sigma_2} \sum_{j=1}^{n_i} (y_{ij1} - \boldsymbol{x}_{ij1}^\top \boldsymbol{\beta}_1 - \boldsymbol{z}_{ij1}^\top \boldsymbol{b}_{i1})^2 \Big\} \times \\
& \frac{1}{\sqrt{2\pi\sigma_2}} \exp\Big[-\frac{1}{2\sigma_2} \sum_{j=1}^{n_i} \big\{ y_{ij2} - \boldsymbol{x}_{ij2}^\top \boldsymbol{\beta}_2 - \boldsymbol{z}_{ij2}^\top \boldsymbol{b}_{i2} - \xi(y_{ij1} - \boldsymbol{x}_{ij1}^\top \boldsymbol{\beta}_1 - \boldsymbol{z}_{ij1}^\top \boldsymbol{b}_{i1}) \big\}^2 \Big].
\end{aligned}
$$

It can be seen that the full conditional posterior distribution of the random effects for $\boldsymbol{b}_{i1}$ is

$$
\begin{aligned}
p(\boldsymbol{b}_{i1} \mid .) \propto\, & p(T_i, \delta_i \mid \boldsymbol{b}_{i1}, \boldsymbol{b}_{i2}, \boldsymbol{\beta}_1, \boldsymbol{\beta}_2, \boldsymbol{\theta}_s) \times \\
& p(\boldsymbol{y}_{i1} \mid \boldsymbol{b}_{i1}, \boldsymbol{\theta}_{y1}) p(\boldsymbol{y}_{i2} \mid \boldsymbol{b}_{i1}, \boldsymbol{b}_{i2}, \boldsymbol{\theta}_{y1}, \boldsymbol{\theta}_{y2}) \times \\
& p(\boldsymbol{b}_{i1} \mid \boldsymbol{\theta}_{y_1}).
\end{aligned}
$$

Thus, the observed values for the second longitudinal outcome $\boldsymbol{y}_{i2}$ appear also in the specification of the posterior distribution of the random effects of the first longitudinal outcome $\boldsymbol{b}_{i1}$. Therefore, the estimation process cannot be entirely separated for the two longitudinal outcomes.

1.1 Priors

We used uninformative normal priors for the β, γ, α, and ϕ_{sk} parameters, as well as for the parameter ξ. For the variance-covariance matrices of the random effects (Σ_{b1}, Σ_{b2}) and for the variance of the error terms of the longitudinal outcomes (σ_1^2, σ_2^2), we respectively used inverse Wishart and gamma priors. The JAGS software was used to analyze the models, using Gibbs sampling for the MCMC methods.

1.2 Code

```
model{
 for (i in 1:N) {

   # Longitudinal Part
   # N is the number of subjects
   # offset is used to distinguish the repeated measurements for each subject
   # y1 and y2 denote the observed longitudinal measurements for the first
   # and second longitudinal outcome
   # b1 and b2 are the random effects
   # Z1 and Z2 are the design matrices for the random effects
   # Note: we use hierarchical centering; for the random effects we sample from
   # a distribution where the fixed effects determine its mean

       for (j in offset[i]:(offset[i+1] - 1)) {
         muy1[j] <- inprod(b1[i, 1:ncZ1], Z1[j, 1:ncZ1])
         y1[j] ~ dnorm(muy1[j], tau1)
         muy2[j] <- inprod(b2[i, 1:ncZ2], Z2[j, 1:ncZ2]) + xi * muy1[j]
```

```
      y2[j] ~ dnorm(muy2[j], tau2)
    }

# Survival Part
# gammasP and gammasD denote the baseline covariates of the survival models
# for open-label medication (Progression) and dropout
# WP and WD are the design matrices for the survival models
# Bs.gammasP and Bs.gammasD are the B-spline coefficients for the baseline
# hazard
# W2P and W2D are the design matrices for the baseline hazards

etaBaselineP[i] <- inprod(gammasP[1:ncWP], WP[i, 1:ncWP])
etaBaselineD[i] <- inprod(gammasD[1:ncWD], WD[i, 1:ncWD])
log.h0.TP[i]    <- inprod(Bs.gammasP[1:ncW2P], W2P[i, 1:ncW2P])
log.h0.TD[i]    <- inprod(Bs.gammasD[1:ncW2D], W2D[i, 1:ncW2D])

# Ztime1P and Ztime1D are the design matrices for the random effects
# defined at the event timepoints

f.T1P[i] <- inprod(b1[i, 1:ncZ1], Ztime1P[i, 1:ncZ1])
f.T1D[i] <- inprod(b1[i, 1:ncZ1], Ztime1D[i, 1:ncZ1])
f.T2P[i] <- inprod(b2[i, 1:ncZ2], Ztime2P[i, 1:ncZ2]) + xi * f.T1P[i]
f.T2D[i] <- inprod(b2[i, 1:ncZ2], Ztime2D[i, 1:ncZ2]) + xi * f.T1D[i]
log.hazardP[i] <- log.h0.TP[i] + etaBaselineP[i] + alphasP1 * f.T1P[i] +
                  alphasP2 * f.T2P[i]
log.hazardD[i] <- log.h0.TD[i] + etaBaselineD[i] + alphasD1 * f.T1D[i] +
                  alphasD2 * f.T2D[i]

# We approximate the integral of the survival function using Gaussian
# quadrature rule, using a 15-point Guass-Konrod quadrature rule
# W2sP and W2sD are the design matrices for the baseline hazards
# with the 15-point Guass-Konrod quadrature rule

for (k in 1:K) {
  log.h0.sP[i, k] <- inprod(Bs.gammasP[1:ncW2P], W2sP[K*(i - 1) + k, 1:ncW2P])
  log.h0.sD[i, k] <- inprod(Bs.gammasD[1:ncW2D], W2sD[K*(i - 1) + k, 1:ncW2D])

  # Zs1P, Zs1D, Zs2P and Zs2P are the design matrices for the random
  # effects with the 15-point Guass-Konrod quadrature rule

  f.s1P[i, k] <- inprod(b1[i, 1:ncZ1], Zs1P[K*(i - 1) + k, 1:ncZ1])
  f.s1D[i, k] <- inprod(b1[i, 1:ncZ1], Zs1D[K*(i - 1) + k, 1:ncZ1])
  f.s2P[i, k] <- inprod(b2[i, 1:ncZ2], Zs2P[K*(i - 1) + k, 1:ncZ2])
                 + xi * f.s1P[i, k]
  f.s2D[i, k] <- inprod(b2[i, 1:ncZ2], Zs2D[K*(i - 1) + k, 1:ncZ2])
                 + xi * f.s1D[i, k]

  # P and D are the observed failure times divided by 2
```

```
    # wk are the prespecified weights

    SurvLongP[i, k] <- wk[k] * exp(log.h0.sP[i, k] + alphasP1 * f.s1P[i, k] +
                       alphasP2 * f.s2P[i, k])
    SurvLongD[i, k] <- wk[k] * exp(log.h0.sD[i, k] + alphasD1 * f.s1D[i, k] +
                       alphasD2 * f.s2D[i, k])
  }

  # To define the density of the survival model we use the zeros trick
  # of WinBUGS, where C is a positive constant

  log.survivalP[i] <- - exp(etaBaselineP[i]) * P[i] * sum(SurvLongP[i, ])
  log.survivalD[i] <- - exp(etaBaselineD[i]) * D[i] * sum(SurvLongD[i, ])
  lambda[i] <- C - ((eventP[i] * log.hazardP[i]) + (eventD[i] * log.hazardD[i])) -
             (log.survivalP[i] + log.survivalD[i])
  zeros[i] ~ dpois(lambda[i])

  # Random Effects Part

  mu_b1[i, 1] <- betas1[1] + betas1[2] * Xtime1D[i,2] + betas1[3] * Xtime1D[i,3] +
                 betas1[4] * Xtime1D[i,4] + betas1[5] * Xtime1D[i,5] +
                 betas1[6] * Xtime1D[i,6] + betas1[7] * Xtime1D[i,7] +
                 betas1[9] * Xtime1D[i,9]
  mu_b1[i, 2] <- betas1[8] + betas1[10] * Xtime1D[i,9]
  mu_b2[i, 1] <- betas2[1] + betas2[2] * Xtime2D[i,2] + betas2[3] * Xtime2D[i,3] +
                 betas2[4] * Xtime2D[i,4] + betas2[5] * Xtime2D[i,5] +
                 betas2[6] * Xtime2D[i,6] + betas2[7] * Xtime2D[i,7] +
                 betas2[9] * Xtime2D[i,9]
  mu_b2[i, 2] <- betas2[8] + betas2[10] * Xtime2D[i,9]
  b1[i, 1:nb1] ~ dmnorm(mu_b1[i, ], inv.D1[, ])
  b2[i, 1:nb2] ~ dmnorm(mu_b2[i, ], inv.D2[, ])
}

#  Priors longitudinal Part

betas1[1:ncX1] ~ dmnorm(priorMean.betas1[], priorTau.betas1[, ])
betas2[1:ncX2] ~ dmnorm(priorMean.betas2[], priorTau.betas2[, ])

xi ~ dnorm(priorMean.xi, priorTau.xi)
tau1 ~ dgamma(priorA.tau1, priorB.tau1)
tau2 ~ dgamma(priorA.tau2, priorB.tau2)

# Priors survival Part

gammasP[1:ncWP] ~ dmnorm(priorMean.gammas[], priorTau.gammas[, ])
gammasD[1:ncWD] ~ dmnorm(priorMean.gammas[], priorTau.gammas[, ])
alphasP1 ~ dnorm(priorMean.alphas, priorTau.alphas)
alphasD1 ~ dnorm(priorMean.alphas, priorTau.alphas)
```

```
  alphasP2 ~ dnorm(priorMean.alphas, priorTau.alphas)
  alphasD2 ~ dnorm(priorMean.alphas, priorTau.alphas)
  Bs.gammasP[1:ncW2P] ~ dmnorm(priorMean.Bs.gammas[], priorTau.Bs.gammas[, ])
  Bs.gammasD[1:ncW2D] ~ dmnorm(priorMean.Bs.gammas[], priorTau.Bs.gammas[, ])

  # Random Effects Part

  inv.D1[1:nb1, 1:nb1] ~ dwish(priorR.D1[, ], priorK.D1)
  inv.D2[1:nb2, 1:nb2] ~ dwish(priorR.D2[, ], priorK.D2)
}
```

Table 1: Results of simulation study 3 based on 200 simulated datasets.

	Values used for simulation based on serial mediator model	Mean values estimated by parallel mediator model
β_{10}	0.070	0.066
β_{11}	-0.250	-0.251
β_{12}	0.120	0.120
β_{20}	0.150	0.116
β_{21}	0.450	0.577
β_{22}	-0.100	-0.160
γ_{11}	-0.200	-0.231
γ_{21}	0.100	0.085
α_{11}	-0.600	-0.600
α_{21}	-0.100	-0.096
α_{12}	0.400	0.391
α_{22}	0.100	0.093
ξ	-0.500	-

2 Extra simulation studies

2.1 Simulation study 3

We performed an additional simulation study to compare the existing multivariate joint model, i.e., the parallel multiple mediator model, with the proposed method, i.e., the serial multiple mediator model. The data were simulated based on the proposed model, using the same settings as in simulation studies 1 and 2.

Figure 1 shows the average overall time-varying intervention effect estimated by the parallel and serial multiple mediator model. As expected, the average overall intervention effect as estimated by these models are equal and very close to the true values.

Table 1 shows the estimates for the path coefficients for the parallel mediator model. The estimates as estimated by the parallel multiple mediator model are relatively close to the values used in the simulation, except for the estimates of β_{20}, β_{21}, and β_{22}. This can be expected as their interpretability in both models is fundamentally different. In the parallel multiple mediator model, the entire intervention effect on the second longitudinal outcome is captured by β_{21} and β_{22}, while in the serial multiple mediator model also part of this intervention effect goes through the first longitudinal outcome. Therefore, their estimates cannot be compared directly, and these differences should not be interpreted as bias in the parallel multiple mediator model.

Another important difference between the models is that the serial multiple mediator model in addition models the dependence between the longitudinal outcomes, contributing to the understanding of the underlying biological process. For the data considered in this manuscript, the estimated size of this additional coefficient (ξ) suggests that there is indeed dependence between the longitudinal brain imaging outcome and the memory impairment ratings.

2.2 Simulation study 4

We conducted an additional simulation study to investigate the robustness of the direct and indirect effects on a setting similar to the real example used. Specifically, we investigated the bias when

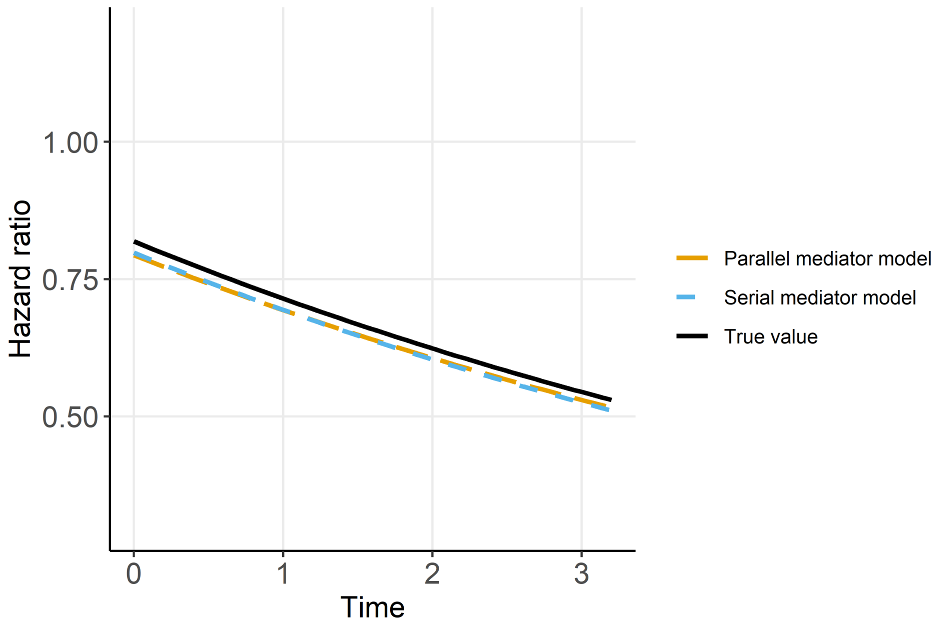

Figure 1: Average time-varying overall intervention effect based on 200 simulated datasets estimated by the parallel and serial mediator model (simulation study 3). The solid black line denotes the true overall intervention effect

the model for the first longitudinal outcome (i.e., linear predictor) includes a mild quadratic fixed (β_{12}) and quadratic random effect for time in the underlying data mechanism, but when these are not taken into account. We used the following parameters: $\xi = -0.5$, $\beta_{10} = 0.07$, $\beta_{11} = -0.40$, $\beta_{12} = 0.07$, $\beta_{13} = 0.12$, $\beta_{20} = 0.15$, $\beta_{21} = 0.45$, $\beta_{22} = -0.10$, $\gamma_{11} = -0.2$, $\gamma_{21} = 0.1$, $\alpha_{11} = -0.4$, $\alpha_{12} = 0.4$, $\alpha_{21} = -0.1$, $\alpha_{22} = 0.1$. Apart from β_{13}, which now denotes the interaction of treatment by time, the other parameters can be interpreted as before. Figure 2 compares the estimated versus the true effects for the direct and indirect effects. As can be seen, the bias is slight.

3 Sensitivity analysis

To evaluate the model goodness-of-fit for the data used in this manuscript, we investigated the model's fit using several figures, including the fitted versus predicted trajectories for different subjects and QQ and residual plots. We concluded that the fit is satisfactory but that the fit for hippocampal volume is better than for NTB memory domain. For example, Figures 3 and 4 show the fitted versus the predicted trajectories for hippocampal volume and NTB memory domain for five randomly selected subjects.

We explored whether we could improve the model fit by using a quadratic model for NTB memory (also because more measurements are performed for NTB memory than for hippocampal volume). For this purpose, we extended the model for $m_{i2}(t)$ with both a fixed and random quadratic effect for time. Table 2 compares the results of the original linear model with the results of the quadratic model. Apart from β_{23}, which is smaller for the quadratic model, the results are very comparable

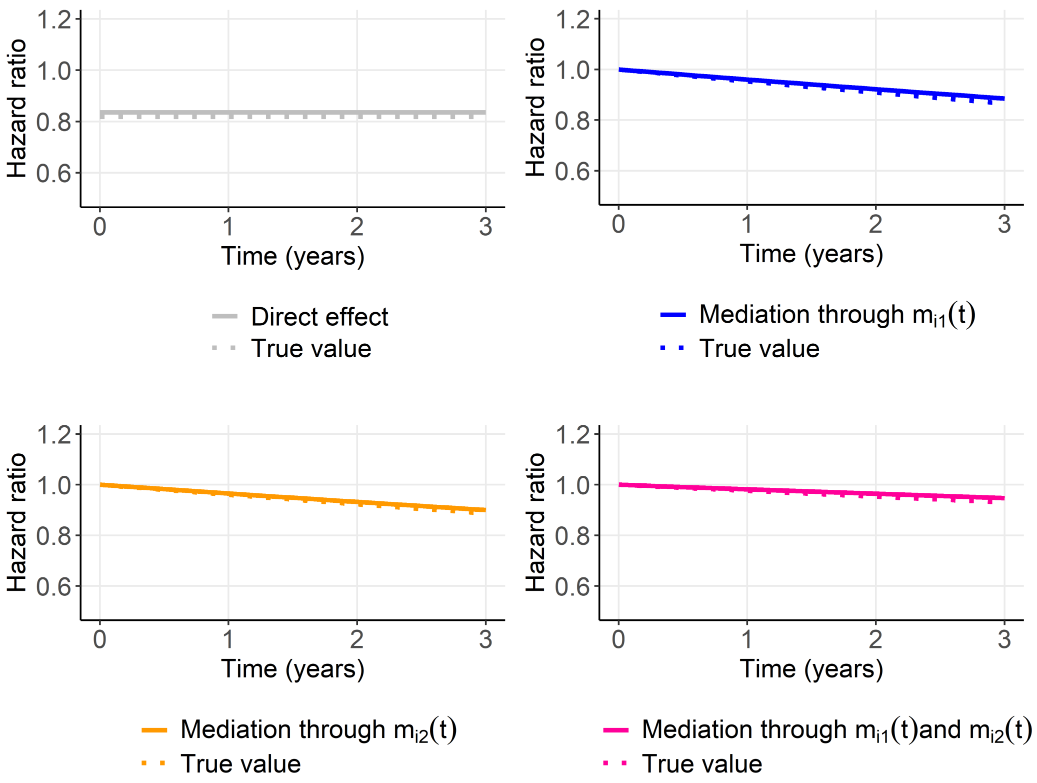

Figure 2: Estimated direct and indirect effects based on 200 simulated datasets (simulation study 4). The dotted lines denote the true effects

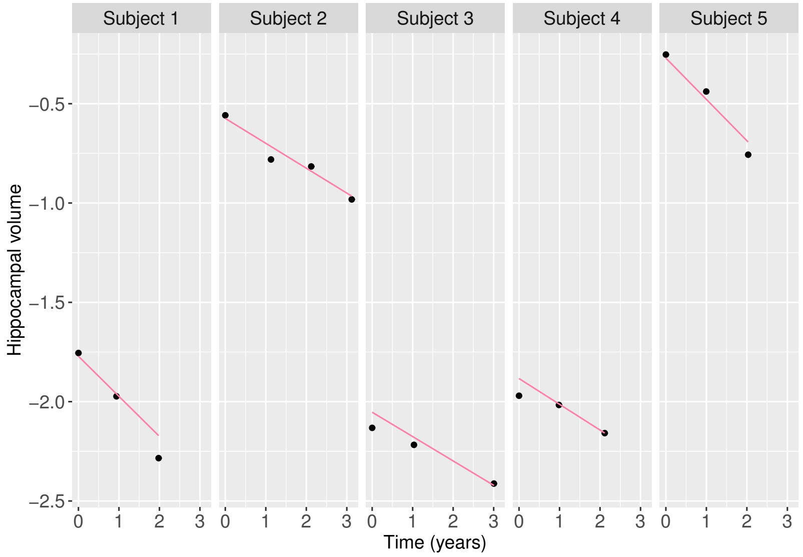

Figure 3: Estimated versus predicted trajectories for hippocampal volume for five randomly selected subjects

for the two different models. Note that the DIC, which is an estimate of the predictive error, is better (lower is better) for the linear model. Figure 5 also shows the fitted versus predicted values for the quadratic model for the same five subjects as before. Figure 6 also compares the QQplots for the linear and quadratic model. We conclude that the quadratic model is no real improvement.

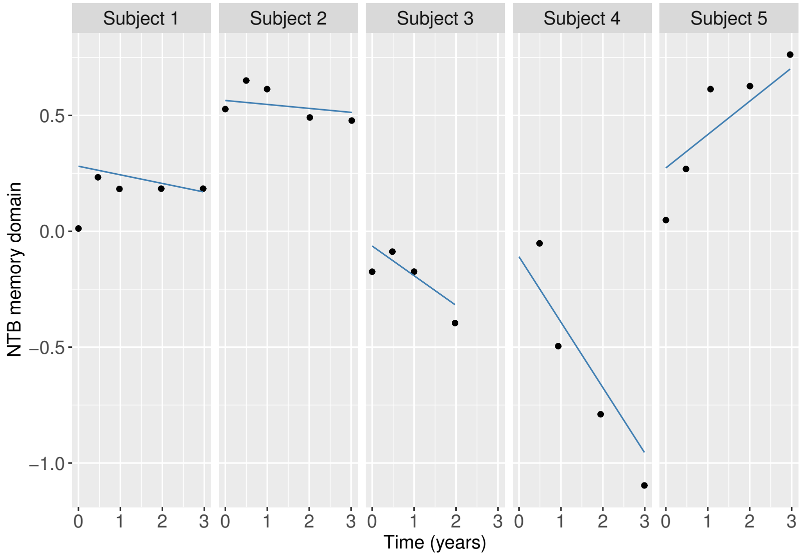

Figure 4: Estimated versus predicted trajectories for NTB memory domain for five randomly selected subjects

Table 2: Results of the original linear and the quadratic model.

	Linear model Mean (SD)	Quadratic model Mean (SD)
**Longitudinal process (Hippocampal volume)**		
Time β_{11}	-0.183 (0.021)	-0.183 (0.020)
Intervention β_{12}	-0.064 (0.118)	-0.060 (0.119)
Intervention $\times$ *time* β_{13}	0.071 (0.030)	0.070 (0.030)
**Longitudinal process (NTB memory domain)**		
Time β_{21}	-0.088 (0.034)	0.024 (0.062)
Time^2 β_{21}	-	-0.041 (0.024)
Intervention β_{22}	0.060 (0.091)	0.068 (0.092)
Intervention $\times$ *time* β_{23}	0.051 (0.046)	0.036 (0.057)
ξ	0.354 (0.057)	0.350 (0.054)
**Survival process (Open-label medication)**		
γ_{11}	0.153 (0.210)	0.124 (0.227)
α_{11}	-0.416 (0.151)	-0.469 (0.157)
α_{12}	-1.073 (0.166)	-1.034 (0.193)
**Survival process (Dropout)**		
γ_{21}	-0.241 (0.179)	-0.263 (0.176)
α_{21}	0.047 (0.142)	0.025 (0.137)
α_{22}	-0.242 (0.136)	-0.207 (0.130)
**DIC**	6430.72	6731.06

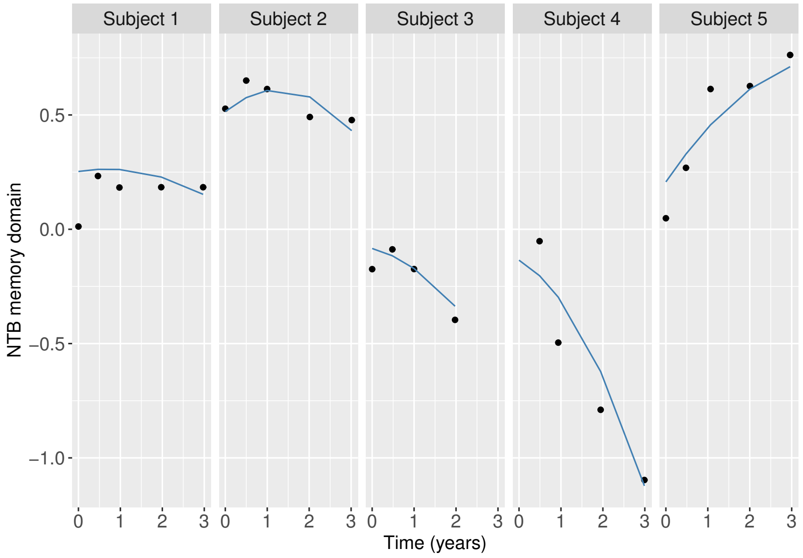

Figure 5: Estimated versus predicted trajectories for NTB memory domain using the quadratic model for five randomly selected subjects

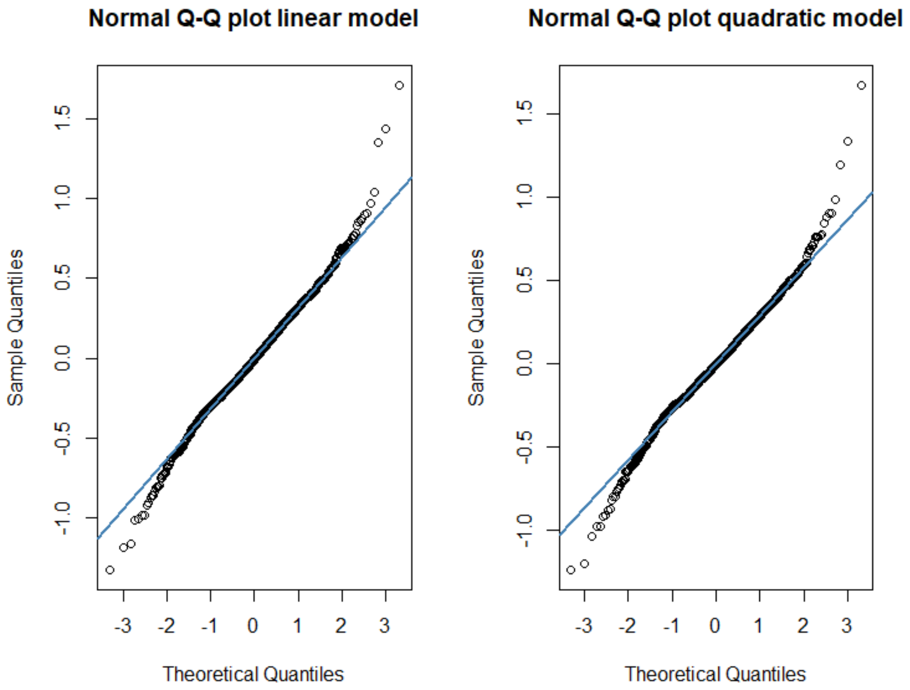

Figure 6: QQplots for the original linear and quadratic model

4 Suppplementary Figures and Tables

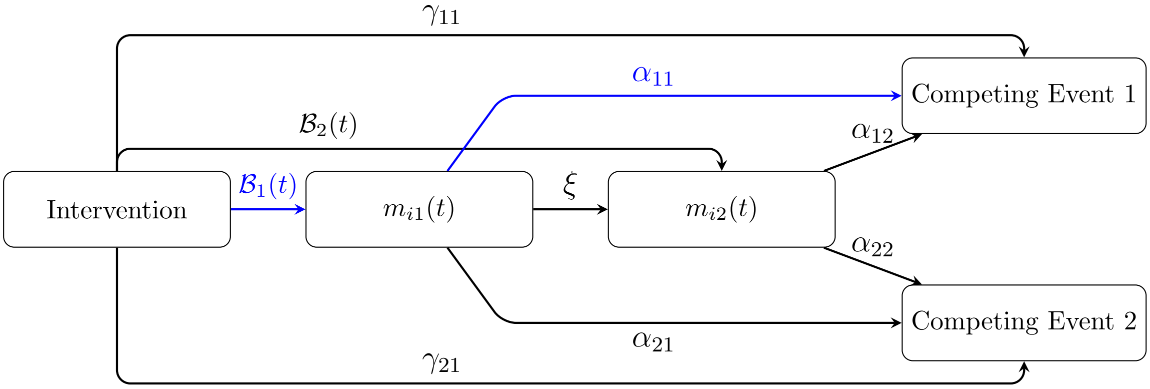

(a) Mediation of the intervention on Competing Event 1 through the first longitudinal outcome

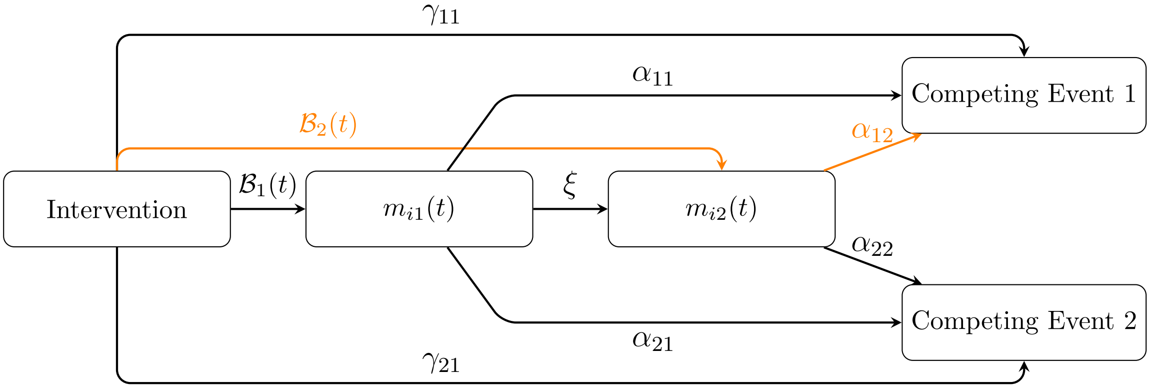

(b) Mediation of the intervention on Competing Event 1 through the second longitudinal outcome

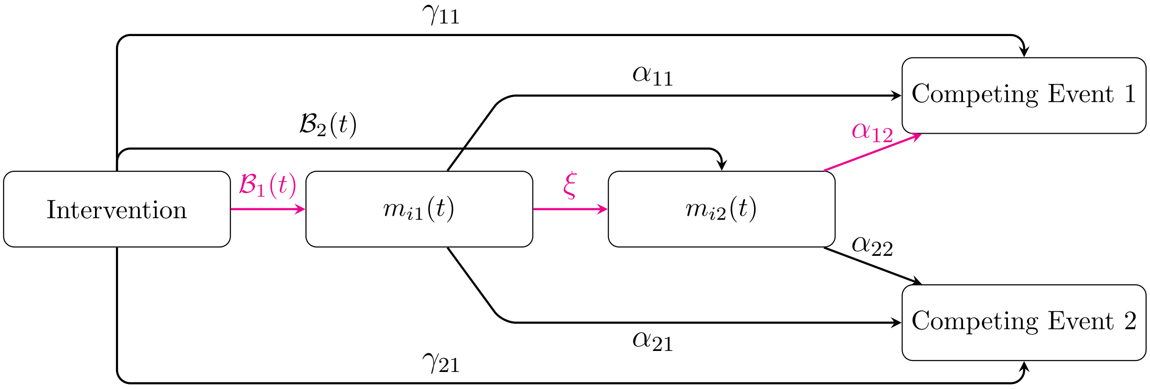

(c) Mediation of the intervention on Competing Event 1 through the first and second longitudinal outcome

Figure 7: The three different indirect paths that link the intervention to the first competing event

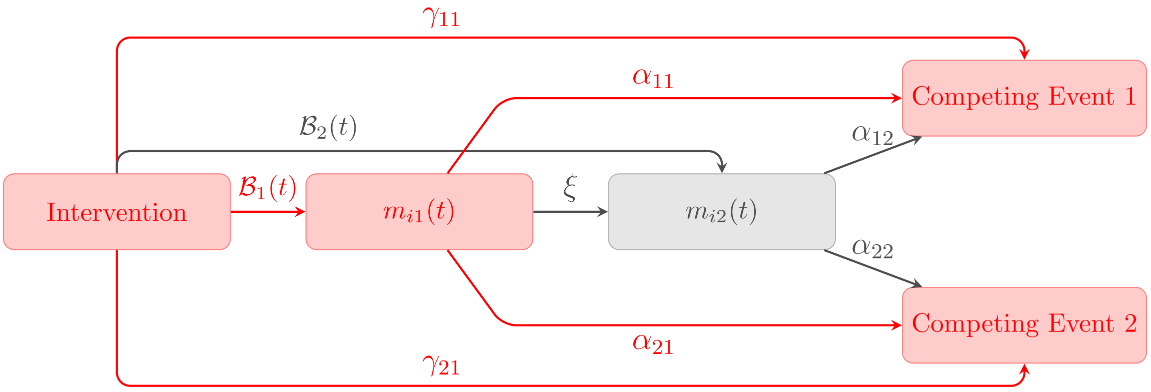

Figure 8: Visualisation of the data analysis step in simulation study 2

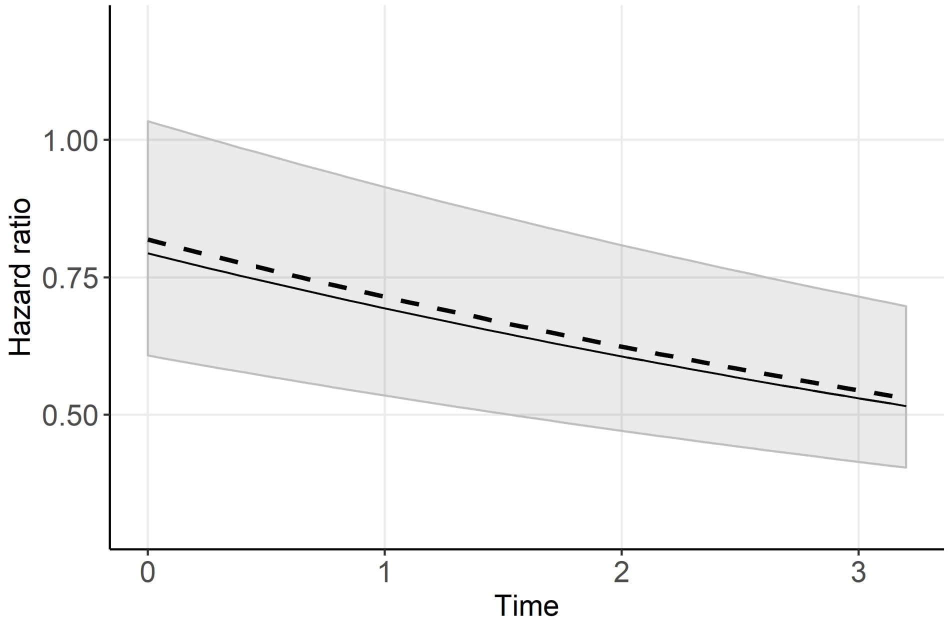

Figure 9: Average time-varying overall intervention effect (solid line) based on 200 simulated datasets with corresponding 95 percentile confidence interval for simulation study 1. The dashed line denotes the true overall intervention effect

Table 3: Posterior means, standard deviation, and the 95% credibility intervals for the multivariate joint model using left hippocampal volume and NTB memory domain as longitudinal outcomes (full results)

	Mean	SD	95% credibility interval
Longitudinal process (Left hippocampal volume)			
β_{10}	-1.149	0.813	-2.749 to 0.464
β_{11}	-0.183	0.021	-0.224 to -0.143
β_{12} $\big(\boldsymbol{\mathcal{B}}_1(t)\big)$	-0.064	0.118	-0.296 to 0.166
β_{13} $\big(\boldsymbol{\mathcal{B}}_1(t)\big)$	0.071	0.030	0.011 to 0.129
β_{14}	0.051	0.030	-0.010 to 0.111
$\beta_{15,1}$	-0.408	0.156	-0.714 to -0.102
$\beta_{15,2}$	-0.096	0.396	-0.884 to 0.688
$\beta_{15,3}$	-0.438	0.194	-0.816 to -0.056
$\beta_{15,4}$	-0.656	0.192	-1.028 to -0.286
$\beta_{15,5}$	0.715	0.193	0.336 to 1.093
Longitudinal process (NTB memory domain)			
β_{20}	-4.122	0.615	-5.333 to -2.918
β_{21}	-0.088	0.034	-0.155 to -0.022
β_{22} $\big(\boldsymbol{\mathcal{B}}_2(t)\big)$	0.060	0.091	-0.119 to 0.239
β_{23} $\big(\boldsymbol{\mathcal{B}}_2(t)\big)$	0.051	0.046	-0.038 to 0.140
β_{24}	0.162	0.023	0.117 to 0.207
$\beta_{25,1}$	-0.389	0.130	-0.645 to -0.133
$\beta_{25,2}$	-0.337	0.196	-0.722 to 0.048
$\beta_{25,3}$	-0.660	0.157	-0.967 to -0.352
$\beta_{25,4}$	-0.625	0.149	-0.914 to -0.333
$\beta_{25,5}$	-0.048	0.160	-0.362 to 0.267
ξ	0.354	0.057	0.244 to 0.465
Survival process (Open-label medication)			
α_{11}	-0.416	0.151	-0.710 to -0.118
α_{12}	-1.073	0.166	-1.409 to -0.758
γ_{11}	0.153	0.210	-0.259 to 0.563
γ_{12}	-0.107	0.036	-0.176 to -0.040
$\gamma_{13,1}$	-0.498	0.328	-1.154 to 0.133
$\gamma_{13,2}$	0.162	0.430	-0.697 to 1.007
$\gamma_{13,3}$	-0.410	0.395	-1.216 to 0.340
$\gamma_{13,4}$	0.071	0.317	-0.564 to 0.680
$\gamma_{13,5}$	0.960	0.439	0.101 to 1.789
Survival process (Dropout)			
α_{21}	0.047	0.142	-0.230 to 0.327
α_{22}	-0.242	0.136	-0.509 to 0.025
γ_{21}	-0.241	0.179	-0.597 to 0.114
γ_{22}	-0.122	0.032	-0.189 to -0.061
$\gamma_{23,1}$	-0.060	0.278	-0.612 to 0.479
$\gamma_{23,1}$	0.562	0.283	-0.005 to 1.102
$\gamma_{23,1}$	-0.547	0.403	-1.385 to 0.203
$\gamma_{23,1}$	-0.096	0.336	-0.766 to 0.552
$\gamma_{23,1}$	0.455	0.317	-0.182 to 1.063
